# The alamar blue assay in the context of safety testing of nanomaterials

**DOI:** 10.3389/ftox.2022.981701

**Published:** 2022-09-28

**Authors:** Eleonora Marta Longhin, Naouale El Yamani, Elise Rundén-Pran, Maria Dusinska

**Affiliations:** Health Effects Laboratory, Department for Environmental Chemistry, NILU-Norwegian Institute for Air Research, Kjeller, Norway

**Keywords:** alamar blue, cytotoxicity, nanoparticles, nanomaterials, viability, cellular metabolic activity

## Abstract

The Alamar Blue (AB) assay is widely used to investigate cytotoxicity, cell proliferation and cellular metabolic activity within different fields of toxicology. The use of the assay with nanomaterials (NMs) entails specific aspects including the potential interference of NMs with the test. The procedure of the AB assay applied for testing NMs is described in detail and step-by-step, from NM preparation, cell exposure, inclusion of interference controls, to the analysis and interpretation of the results. Provided that the proper procedure is followed, and relevant controls are included, the AB assay is a reliable and high throughput test to evaluate the cytotoxicity/proliferation/metabolic response of cells exposed to NMs.

## Graphical Abstract

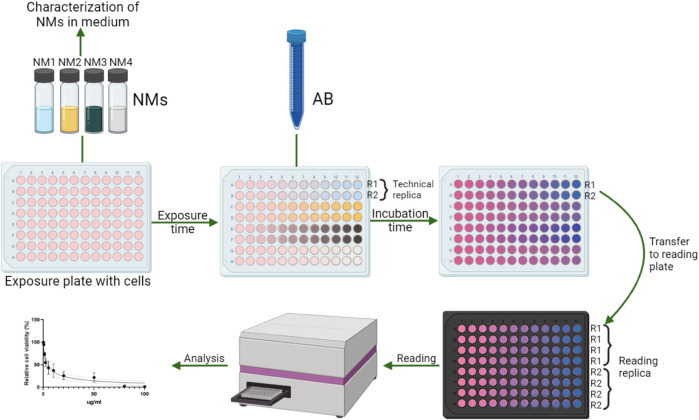

## 1 Introduction

Cytotoxicity is one of the main endpoints to be assessed in any toxicological investigation. There is a wide range of methods that can be used to investigate the cytotoxic effects of chemicals and other test substances, including nanomaterials (NMs). These methods are based on diverse principles and cell functions, e.g., membrane integrity (assays such as trypan blue exclusion, neutral red uptake, LDH release), relative cell growth (measuring the number of cells in the population, reflecting cell death together with changes in cell proliferation), ability to survive and form colonies [colony forming efficiency assay (CFE)], and cellular metabolic competence (Riss et al., 2016; 2019; Méry et al., 2017). This last class of methods uses the cellular metabolic activity to measure viability or proliferation in a cell population. Metabolically active cells maintain a reducing environment within their cytosol. This is taken advantage of through the use of colorimetric or fluorometric redox indicators, and their conversion that can be measured spectrophotometrically. Together with tetrazolium salt-based assays such as MTT and WST-1, the Alamar Blue (AB) assay is one of these metabolism-based methods. Since its release in 1993, the AB assay has become widely used to investigate *in vitro* the cytotoxicity of various test compounds, and the proliferation of cell lines, bacteria and fungi (Fields and Lancaster, 1993; Ahmed et al., 1994). AB is based on the fluorometric redox indicator resazurin (7-hydroxy-3H-phenoxazin-3-one 10-oxide), a blue-colored non-fluorescent compound. After intracellular uptake, the oxidized resazurin is reduced to the fluorescent resorufin (7-hydroxy-3H-phenoxazin-3-one) due to the reducing environment of the cytosol in the cells. The conversion of resazurin to resorufin is mediated by intracellular diaphorases, with NADPH or NADH as reductant (O’brien et al., 2000). Resorufin produces bright red fluorescence, with excitation range of 530–570 nm and emission range of 580–610 nm, that can be quantified (fluorescence intensity) and used as a measure of cell viability. The test can also be read on the basis of the absorbance at 570 nm, using 600 nm as a reference wavelength (the values need to be normalized on the reference wavelength).

Resazurin (and resorufin) is water-soluble, stable in culture medium, non-toxic and permeable through cell membranes, and the AB assay has proved to be robust, simple to perform and relatively cheap, thus presenting many advantages compared with other cell viability and proliferation assays (Rampersad, 2012). As an example, the AB assay has clear advantages with respect to the MTT assay, another common cytotoxicity method based on cellular metabolic activity: 1) First, being water-soluble resorufin is released in the cell culture medium, which can be directly used for measurement. In contrast, the insoluble formazan crystals produced by the conversion of the tetrazolium salt in MTT need to be dissolved by a solubilization step before reading the test. 2) Additionally, the cells used for MTT will thus be destroyed during the solubilization step, while the cells used for AB can be employed for other purposes. 3) AB is non-toxic, while MTT has been reported to be cytotoxic itself (Ghasemi et al., 2021). 4) Finally, AB has been reported to be more sensitive at detecting cytotoxicity than the MTT assay (Hamid et al., 2004).

The AB assay can be used in a high throughput set up, allowing screening of the toxicity of a large number of compounds at the same time (Hamid et al., 2004; OECD, 2018), and it has been widely used within the field of nanotoxicology [287 PubMed search results with keywords: (Alamar Blue) AND (nanomaterials OR nanoparticles)].

For cytotoxicity testing of NMs, interference is an important challenge especially in relation to colorimetric and fluorescent test methods. NMs can in general interfere with toxicological tests at different levels, from the assay’s chemical reactions to the test readout (Rampersad, 2012; Guadagnini et al., 2015). As an example, spectroscopic analyses have highlighted interactions (indicated by reduction of absorption/fluorescent emission) of single walled carbon nanotubes (SWCNT) with several dyes used for cytotoxicity investigations, including Neutral Red, MTT, WST-1, and also AB, which was found to be the most sensitive and reproducible method (Casey et al., 2007; Davoren et al., 2007). Interference issues might account for the inconsistency sometimes found in the responses obtained with the different cytotoxicity methods; therefore the verification of the results by the use of at least two methods is recommended (Worle-Knirsch et al., 2006; Dusinska et al., 2015, 2017). Repeated washing steps should be performed after exposure to remove as many particles as possible. However, internalized particles or particles adhering to the cell surface will not be removed after washing (Davoren et al., 2007). To properly address this issue, appropriate controls to check for interference should always be included in the experiments.

General considerations and potential pitfalls of the AB assay have been previously reported in the literature (Rampersad, 2012). Here we thoroughly describe the AB procedure applied to NMs, present examples of results obtained with various types of NMs and discuss possible interpretation of the results. The method here described has been refined during the many years of NM-related research in our laboratories, and within the H2020 project RiskGONE, whose core aim is to evaluate the suitability of various *in vitro* tests for reliable hazard assessment of NMs, and to deliver sound protocols that have been adapted for use with NMs.

## 2 Materials and equipment

### 2.1 Reagents and materials

AB can be purchased from different providers as a ready-to-use solution, although a resazurin sodium salt is also available. The procedure below is for the ready-to-use solution, and it was developed in our laboratory based on the manufacturer’s instructions, with the addition of some refined steps and specific measures for testing NMs. Further adaptations might be needed by the operator according to the product purchased. The manufacturer’s instructions should always be the basis on which to apply the NM-specific measures.

Over the years, several NMs have been tested with the AB assay in our laboratory within different projects. Here we mostly refer to the work performed under the ongoing H2020 RiskGONE project, where the AB assay was critically examined for its suitability for testing NMs. To this end, different NMs were selected. As an example, here we present the results obtained with TiO_2_-based NMs JRCNM01005a [European Registry of Materials: ERM00000064 (van Rijn et al., 2021)] and ZnO NMs from Sigma Aldrich (supplier code 721077, ERM00000063). Results obtained on NMs tested within the EuroNanoMed II project GEMNS (GEMNS-IVA1) are also reported. More information on the NMs is reported in Table 1.

**TABLE 1:** Information on the NMs used.

NMs	Provider and code	European registry of materials (van Rijn et al., 2021)	Particle size according to the provider
TiO_2_	JRCNM01005a	ERM00000064	15–24 nm
ZnO	Sigma Aldrich, 721077	ERM00000063	<100 nm (TEM)
GEMNS-IVA1	Graphene-Encapsulated Magnetic Nanoparticles functionalized with polymers (PEI, 25 kDa) and decorated with human IgG	NA	Full characterization available in (Kasprzak et al., 2016)

### 2.2 Equipment

Equipment needed for the AB assay include a laminar flow hood, light microscope, automated cell counter/Bürker chamber, pipettes, CO_2_ incubator, refrigerator, water bath, vortex, autoclave.

For reading a spectrometer, fluorometer, or plate reader for higher throughput, with filters or monochromator to read fluorescence within excitation range of 530–570 nm and emission range of 580–610 nm, or absorbance at 570 and 600 nm are needed for reading. In this manuscript a microplate reader FLUO star OPTIMA was used to read fluorescence (excitation 530 nm, emission 590 nm).

## 3 Methods

### 3.1 Cell culture conditions

The AB test can be applied to both adherent cells and cells in suspension, as well as co-cultures and 3D advanced models. The cells are cultivated according to the model’s specific needs.

Different exposure plates can be used. The use of 96 well plates is convenient to increase the throughput of the method, especially useful for toxicity screening.

The number of cells to be seeded for exposure is an important parameter to consider, and it should be adjusted based on the cell type used. It has been reported that the cell density at the moment of exposure (confluency) can affect the sensitivity of the cells to NMs; i.e., lower EC50 values are observed when fewer cells are exposed, in tetrazolium based assays (Geys et al., 2010; Heng et al., 2011; Elliott et al., 2017). This has been observed also in our laboratory with the AB assay (the data are not shown, as further investigations on this topic are needed). According to the OECD Guidance Document on Good *In Vitro* Method Practices (GIVIMP) (OECD, 2018) a fixed and pre-determined seeding density should be used to improve consistency across experiments, and can contribute rather than an estimation of the cell confluency that is prone to error and contribute to variability in baseline cell physiology. Thus, the seeding density is a parameter that needs to be harmonized within and among laboratories. It is generally recommended that the cells should be used in the exponential growth phase (Rampersad, 2012).

The cell lines and conditions used in this study are reported below.

#### 3.1.1 Adherent cells

The human lung epithelial cell line A549 was maintained in DMEM medium supplemented with 9% FBS and 1% penicillin/streptomycin, in an incubator at 37°C, 5% CO_2_; the day before exposure, 1.5 × 10^4^ cells/well were seeded in a 96 well plate. The human bronchial epithelium cell line BEAS-2B was cultivated in LHC-9 medium without supplements; the day before exposure, 2.0 × 10^4^ cells/well were seeded in a 96 well plate.

#### 3.1.2 Suspension cells

The human lymphoblast cell line TK6 (suspension cells) was maintained in RPMI medium supplemented with 9% HS and 1% penicillin/streptomycin; the same day of exposure, 1.5 × 10^4^ cells/well were transferred in a 96 well plate.

N.B. Cells in suspension can be transferred to the exposure plate on the same day of exposure. Cells must be seeded in half of the medium volume that will be used for exposure, e.g., if 200 µl of medium per well are used in the 96 well format plate as final exposure volume, the correct number of cells should be transferred in 100 µl of medium per well.

### 3.2 Nanomaterial dispersion and characterization

The NMs used for the test should be properly dispersed and characterized. Different approaches and methods are available to address the NM dispersion. Not all NMs respond equally to the same handling. In this work we mainly used the protocol described in (Deloid et al., 2017). When satisfactory dispersion was not obtained (particle size and size distribution by DLS analyses vs. expected particle size based on the provider’s declaration), the NANOGENOTOX protocol was tested and applied if better results were observed.

Proper NM characterization should always accompany any toxicological study.

### 3.3 Exposure conditions and treatment with test substance and controls

For exposure of cells to the test substance, it is good practice to have technical replicates within the same experiment e.g., at least two wells exposed to the same treatment. At least three independent experiments should be performed.

A negative control (NC) and a positive control (PC) must always be included, i.e., cells unexposed to the test substance (maintained in the cell culture medium) and cells exposed to a known cytotoxic agent, respectively. This allows assessment of the performance of the assay. A possible positive control recommended for the AB assay, and used in the experiments here reported, is chlorpromazine at 50 µM. Other agents giving a positive response, to be adapted to the cell model used, can be considered. Specific considerations and guidance on the selection of proper positive control materials for *in vitro* assays can be found in the literature (Petersen et al., 2021).

A range of NM concentrations should be included to establish a concentration response curve. A minimum of 3 concentrations in addition to the negative control sample should be considered. Cell-particle interaction should be assessed, i.e., NM deposition or internalization.

N.B. Cells in suspension must be exposed by adding twice concentrated exposure doses at ratio 1:1 to the seeding medium volume; e.g., if 200 µl of medium per well are used in the 96 well format plate as final exposure volume, 100 µl per well of 2x concentrated exposure suspensions must be added to 100 µl of plated cells.

Interference controls for NMs must be included. These consist of NMs in cell culture medium in wells without cells (only medium with NMs). The highest concentration tested for the NMs should be included as a minimum condition; however other test concentrations could be added in an optimal situation. These controls will be incubated for the same time as the exposed cell samples.

### 3.4 Alamar Blue preparation

The AB storage conditions should be followed according to the manufacturer’s instructions. In general, the AB solution should be stored in the dark and protected from light during the performance of the assay, since the compound is light sensitive. The AB solution can be stored at room temperature or at 4°C (for extended shelf life); if stored cold, it should be equilibrated to room temperature before use.

The solution should be slightly shaken to ensure all components are completely in solution before use.

N.B. Clogging or precipitates of AB can be sometimes observed. Clogging in the staining solution will affect the results, making them unreliable. It is important to always check that the staining solution is free of precipitates. These can be removed by filtering the AB solution or the staining solution (AB and medium) before mixing it with the medium (syringe-filter through a 0.2 or 0.45 µm pore filter).

### 3.5 Alamar Blue incubation

As the AB assay is very sensitive, precise pipetting is important in the following steps, to obtain reliable results. Do not leave any residue of PBS or medium after washing, to avoid diluting the staining solution. Pipette the exact volume of the staining solution into the wells. Uneven volume pipetting will render results unreliable. Special attention should be given when using multichannel pipettes. With adherent cells, gently pipet along the wall of the well, to avoid detaching the cells by harsh pipetting.

#### 3.5.1 Adherent cells

- At the end of the exposure to the test substance or NMs, prepare the staining solution by adding 10% AB to fresh cell culture medium pre-heated to 37°C.
- Exposed cell samples: Remove the exposure medium and wash the cells twice in PBS or medium. Add the staining solution, e.g., 200 µl in the 96 well plate.
- Interference controls for NMs: add pure AB to the interference controls to a final concentration of 10%; e.g., if 200 µl of medium are used in the 96 well plate, mix the medium in the interference control wells, discard 20 µl of medium and replace with 20 µl of pure AB (alternatively add 22 µl of pure AB to 200 µl of medium). Mix thoroughly again.

#### 3.5.2 Suspension cells

- At the end of the exposure to the test substance or NMs, add pure AB to the exposed cells samples and interference controls to a final concentration of 10%, e.g., if 200 µl of medium are used in the 96 well plate, mix the medium in the wells, discard 20 µl of medium and replace with 20 µl of pure AB (alternatively add 22 µl of pure AB to 200 µl of medium). Mix thoroughly again.

#### 3.5.3 All cell types

Always include blank control sample: add the staining solution (10% AB in cell culture medium) in empty wells (wells without cells). Use the same volume as the other samples, e.g., 200 µl in the 96 well format plate.

After adding AB, incubate the plate for 1–4 h at 37°C, 5% CO_2_, until a change in AB color can be observed. A longer incubation time may be used for higher sensitivity. The incubation time depends on several factors which include the cell type (cells with different metabolism convert AB at different speed rate), the cell model (3D models e.g., spheroids might take more time to convert AB with respect to the same cell type in 2D condition, due to the reduced cell surface available, thus reduced AB uptake) and the number of seeded cells (Bonnier et al., 2015). Find the optimal incubation time for the system used and standardize it for further experiments. In our experience, 3 h incubation time is appropriate in most cases.

### 3.6 Reading

At the end of the incubation time, the AB signal can be read in absorbance or fluorescence by a spectrometer or fluorometer, respectively. Fluorescence seems to provide higher test sensitivity compared to absorbance. The staining solution can be transferred to reading supports such as cuvettes or, to increase the throughput of the assay to, e.g., 96-well format reading plates. For reading of the fluorescent signal in microplates, black plates should be used, as in the transparent plates signal interference from the next wells might occur.

To increase the robustness of the results, 3 or 4 reading replicas should be prepared from each sample. For example, if 96 well plates are used for exposure, transfer 40 µl of staining solution (medium) 4 times into 4 different wells of flat bottom 96 well black polystyrene microplates.

N.B. The presence of bubbles in the medium during reading can affect the results, and thus must be avoided. Pipetting when transferring the medium to the reading support must be done with care; the reverse pipetting technique might be of help for this. After pipetting, the presence of bubbles should be checked by visual inspection. There are several ways to remove bubbles, e.g., blowing a gentle stream of air or ethanol vapor over the plate, putting the plate or cuvette under vacuum, or using a 10 µl pipette tip (Petersen et al., 2022). The potential application of a bubble control might be considered (ISO, 2018; Petersen et al., 2022).

The fluorescence signal or absorbance can be read at appropriate wavelengths in the microplate plate reader. For fluorescence, the AB excitation range is 540–570 nm and emission range is 580–610 nm. The AB absorbance can be read at 570 nm, using 600 nm as a reference wavelength. The data are obtained as fluorescence units (FU) or optical density (OD), respectively.

#### 3.6.1 Optional

A centrifugation step can be included before transferring the staining solution to the reading plate/support. Preliminary data in our laboratory suggest that this step could be especially useful when suspension cells are used, or in case of interference of NMs with the test reading (data not shown).

### 3.7 Data analysis

The results of the AB assay can be presented as relative fluorescence (or absorbance) intensity (percentage) of the exposed samples towards that of unexposed cells i.e., the negative control. While results are linear and quantitative for both fluorescence and absorbance, the fluorescence readings provide higher sensitivity.

The values for the fluorescence reading are calculated as described below:

- calculate the average of the reading replicates
- subtract the average of the blank control (samples with AB and without cells) from all the data
- calculate the average of the negative control samples (technical replicate)
- calculate the relative fluorescence intensity as the ratio between the exposed samples and the average of the negative control samples, and express it as a percentage according to the equation:

Relative fluorescence intensity: FU exposed sample/FU average negative controls *100.

The average ±SD (or SEM) of at least 3 independent experiments should be calculated and reported as the final result.

#### 3.7.1 Interference controls for nanomaterials

Interference control samples for NMs (10% AB in medium + NMs without cells) should be compared with the blank control samples (10% AB in medium without cells). This can be done at the level of FU, i.e., no significant difference between FU of interference control samples and FU of blank samples indicating lack of interference. Alternatively, the interference controls can be analyzed as the other samples, i.e., the relative fluorescence intensity can be calculated. In this case the relative fluorescence intensity value obtained for the interference control sample is expected to be around 0%. This last approach has been used for the results here reported.

#### 3.7.2 Historical positive and negative controls

It is highly recommended to build up an historical control database, with both negative as well as positive controls for each cell type and time point investigated. This allows the laboratory to demonstrate the ability to perform the assay consistently, and to show that the cells are capable of picking up positive effects and have reasonably low variability in responses. When reporting the results, it is advisable to show the average and minimum-maximum values of negative/positive historical controls from the last 10–20 experiments performed in the laboratory.

In the AB assay the results are normalized over the NC, so this will always be 100% (Relative fluorescence intensity). Historical PC values from our laboratory (Chlorpromazine 50 µM, on different cell lines and exposure times) are reported in Table 2 as an example (average of the last 10 experiments ±SD, minimum and maximum values).

**TABLE 2:** Historical PC data for Chlorpromazine 50 µM (by cell lines and exposure time). Data are reported as relative fluorescence intensity (%) with respect to the NC (100%).

Cell line, exposure time	Average ±SD	Minimum value	Maximum value
A549, 3 h	3.9 ± 7.7	−3.1	18.5
A549, 24 h	−1.8 ± 3.8	−11.3	1.7
BEAS-2B, 3 h	2.0 ± 9.9	−8.2	20.6
BEAS-2B, 24 h	−1.4 ± 1.7	−4.5	0.2
TK6, 3 h	56.6 ± 15.1	42.5	77.9
TK6, 24 h	4.4 ± 8.9	16.1	−4.9

#### 3.7.3 Data collection templates

Within the RiskGONE project, a data collection template has been developed for AB, to move towards data reporting harmonization and data FAIRness. The template provides a function for automatic calculation of the results from the reported raw data. The template is available upon request through the eNanomapper database, and it will be made publicly available (Jeliazkova et al., in preparation).

### 3.8 Interpretation of the results

The criteria for determining if a test compound is cytotoxic can depend on the application field.

In general, the test substance is considered cytotoxic if all the following conditions occur:

- The signal in the cells treated with the test substance is reduced at least by 20% compared to the negative controls (untreated cells)
- A concentration-related reduced signal is observed
- The results are reproducible, i.e., at least 3 independent experiments confirm the results

The first point reflects the fact that the biological relevance of the results needs to be considered. According to the historical controls, the variability (calculated as standard deviation, SD) between the experiments can account for around 10% of the relative fluorescence intensity calculated with respect to the NC. To ensure the biological relevance of the observed reduction, 2x SD (20%) is selected as a threshold to state that a compound is cytotoxic. Statistical methods are used as an aid in evaluating the test results. However, the statistical significance will not be the only determining factor for cytotoxicity.

A test substance, for which the results do not meet all the above criteria, is considered non-cytotoxic under the assay conditions.

Positive results in an *in vitro* cytotoxicity test indicate that the test substance induces a cytotoxic effect in the cultured cells used. Negative results indicate that, under the test conditions, the test substance does not induce cytotoxicity in the cells used.

#### 3.8.1 Interpretation of the interference controls results

If the FU value of the interference samples significantly deviates from that of the blank sample (or the relative fluorescence intensity deviates from 0%), it means that the NMs interfere with the AB assay and the results obtained are not reliable. Also in this case, the criteria for determining if an interference is present can depend on the application field, and on the criteria applied to determine the cytotoxicity of the test compound. A statistically significant difference between the interference control and the blank could be a sufficient indication of interference.

## 4 Results

**FIGURE 1:**
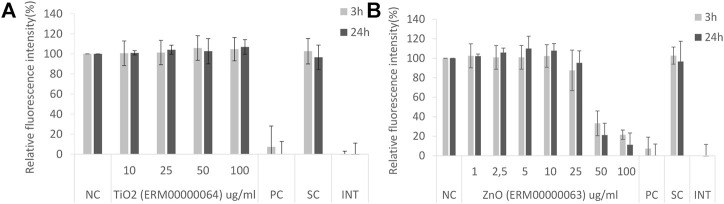
Alamar Blue assay on A549 cells after 3 and 24 exposure to nanomaterials: **(A)** TiO_2_ JRCNM01005a (ERM00000064) and **(B)** ZnO NMs from Sigma Aldrich (supplier code 721077, ERM00000063). NC-negative control, PC-positive control, SC-solvent control, INT-interference control.

Here we report some examples of results obtained with different NMs used in various projects. Within the ongoing H2020 RiskGONE project, the cytotoxicity/cellular metabolic activity of cells exposed to different NMs was analyzed by the AB assay, to evaluate the method’s suitability for testing NMs. Both adherent (A549, Figure 1) and suspension cells (TK6, data not shown) were used to test the assay. The interference controls were also included and the reported value were similar to the blank for all the NMs tested (no interference of the tested NMs with the AB assay was detected, Figure 1).

Non-cytotoxic compounds such as TiO2 ERM00000064 result in a relative fluorescence intensity similar to that of the negative control (100%) as shown in Figure 1A. In the case of a cytotoxic compound such as ZnO ERM00000063 (Figure 1B), a concentration response curve will be obtained, showing the reduction of signal with increasing concentrations of NMs. The shape and the slope of the curve give an indication of the severity of the toxicity of the test substance. From this slope, the EC50 (effective concentration producing 50% of the maximal response) can be calculated.

**FIGURE 2:**
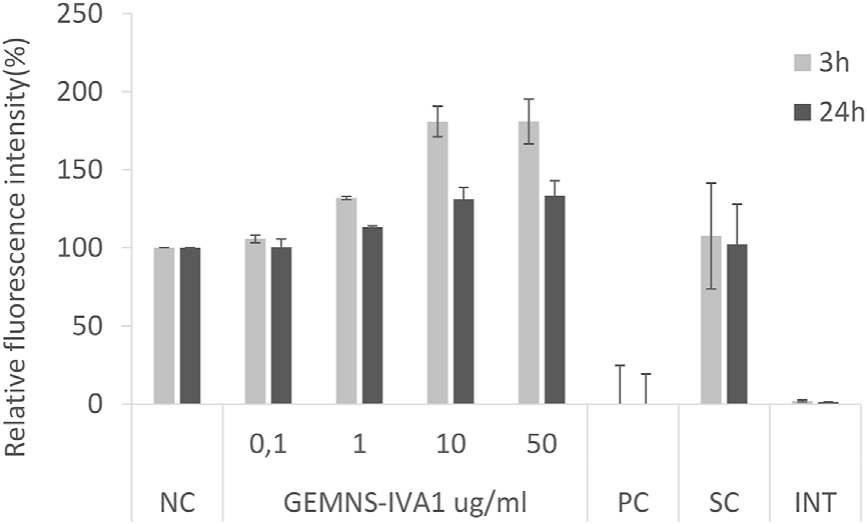
Alamar Blue assay on A549 cells after 3 and 24 exposure to GEMNS-IVA1 nanomaterial. NC-negative control, PC-positive control, SC-solvent control, INT-interference control.

Besides the more common situations reported above, different results can be observed. Interference of NMs with the test could be found, although in our experience it was rarely detected. An increase of the relative fluorescence was observed on a few occasions in the samples exposed to the test substance compared to the negative control. NMs tested within the EuroNanoMed II projects GEMNS highlighted a concentration-dependent increase of the AB signal. This effect was observed in both A549 cells (Figure 2) and BEAS-2B cells (data not shown). In both cases the effect was more evident after 3 h exposure to the NMs, compared to 24 h exposure. An interference effect was ruled out as shown by the interference control (Figure 2).

## 5 Discussion

The AB assay is widely used on mammalian cells and cell lines, bacteria and fungi to establish the relative cytotoxicity of test substances, demonstrating it to be a reliable test. However, there are important aspects (and possible disadvantages) in this assay to take into consideration, the first being that AB is not a direct cell counting technique, and the fluorescence or absorbance signal can be affected by changes both in the number of living cells and in cellular metabolism. The test provides information at the level of the whole cell population, not the single cell, with the assumption that cytotoxicity will determine a reduction in the number of cells, and a lower resorufin signal. Damaged and/or non-viable cells have lower innate metabolic activity and thus generate a proportionally weaker signal than healthy cells. On the other hand, some compounds, including NMs, can increase the metabolism of the cells (Kladko et al., 2021), which will result in a higher AB signal. Besides, alterations of cell proliferation are not always accompanied by cell death. The test substance might influence cell proliferation, either slowing it down or accelerating it, affecting the total number of cells, and thus the test results.

For this reason, caution should be taken in the interpretation of the data, in the sense that the results might not be directly an indication of cytotoxicity, but an effect of the cellular metabolic activity or proliferation. These processes, cytotoxicity, metabolic activity and proliferation, all play together in determining the response of the test, thus making the interpretation of the results more challenging.

Interestingly, it has also been reported that a further reduction of resorufin leads to the formation of colorless and non-fluorescent products. Thus, aberrant results might be generated when healthy cells over-reduce the AB producing a weaker signal compared to less active or dying cells (O’brien et al., 2000).

In this context, the use of multiple assays (at least two cytotoxicity tests) is recommended to reduce false negative/positive results (Worle-Knirsch et al., 2006; Dusinska et al., 2015, Dusinska et al., 2017, Azqueta et al., 2022). Non-colorimetric assays such as the CFE or clonogenic assay, and impedance-based assays represent valuable and interference-free tools to support the cytotoxicity investigation of NMs (Herzog et al., 2007; Cimpan et al., 2013; Rundén-Pran et al., this special collection). Visual (microscopy) evaluation of the status of the cells should also always be performed.

In the use of *in vitro* tests with NMs, one should take into consideration the different behaviors and physico-chemical properties of these materials compared to chemicals in general. Just to mention a few, the capacity of NMs to adsorb other compounds on their surface, and optical properties such as optical density that can interfere with the transmission of light, and in some cases autofluorescence. These properties are in particular relevant when colorimetric or fluorometric test methods are used, such as the AB assay. As this test is based on the development and measurement of fluorescence (or alternatively absorbance), there is a chance for NMs to interfere with the test read-out. In addition, NMs might directly interact with the reagent, altering its structure and affecting the normal reactions that should occur.

It is not the aim of this work to deeply investigate the possible interference of the AB assay with NMs, but rather to revise and adapt the method for application to this class of substances. Interference controls are meant to assess any interference of the NMs with the AB. This can happen at different levels, from interactions with the reagent or product at different steps of the assay, or with the reading as quenching of fluorescence or as a false induced signal, e.g. autofluorescence. In our approach, possible interference is investigated by mixing the NMs with the AB and analyzing the signal. This sample can be analyzed as the other samples, i.e., the relative fluorescence intensity can be calculated. In this case the expected outcome is a null relative fluorescence intensity, similar to the blank samples. A higher signal could indicate e.g., autofluorescence of the NMs analyzed, while a lower value could indicate shading of the fluorescent signal. However, this last condition would be difficult to detect with this setting of interference control, as resazurin alone is not fluorescent. An additional control mixing NMs with the fluorescent resorufin could be considered.

The interpretation of the results obtained from cells exposed to NMs and chemicals in general can sometimes be tricky. On a few occasions we observed an increased relative fluorescent signal in cells exposed to NMs compared to the non-exposed (negative) control, such as is shown here in Figure 2. Possible reasons for this effect could be an increased metabolism of the cells in response to the test substance, or increased proliferation resulting in a higher number of cells. However, the first hypothesis seems more likely in our case, at least for the effect observed at 3 h after exposure, which might be too early to see an increased number of cells. This last option might explain the slight increase of fluorescent signal at 24 h when the cells had time for the cell cycle to be completed.

In general, an interference of the NMs with the test cannot be excluded in case of increased fluorescent signal, e.g. due to NM autofluorescence, or to NMs reacting with the resazurin in the AB and reducing it to the fluorescent resorufin. Strongly reducing NMs may directly reduce resazurin non-enzymatically. Compounds that trigger the release of superoxide can cause reduction of resazurin by superoxide. This may result in a false cytotoxicity outcome. In our case here this seems to be excluded as the interference controls did not show any increased signal.

In case of interference of NMs with the test (or even when suspension cells are used), centrifugation of the samples before reading might be a way to remove the NMs (and cells in suspension). A plate spinner helps in case of the high throughput setting. Another possible solution suggested is to use the interference controls values as background value, and thus subtract them from the correspondent NM-exposed sample value (Guadagnini et al., 2015; Ciappellano et al., 2016). In this case, interference control samples for all the NM concentrations tested must be included, and the relative value subtracted for each concentration.

The AB assay is included as a part of the OECD TG 249 for the RTgill-W1 fish cell line acute toxicity test into 24-well plates. No dedicated standard method with detailed operating procedure is available at the moment, e.g., for the use with other cell lines, for higher throughput format, and for testing with NMs. The work here presented will help the standardization of this test to support sound safety assessment of NMs, by providing a detailed procedure that can be tested among different laboratories. The next steps towards standardization should include a validation of the procedure through interlaboratory testing of specific settings (e.g., selected cell lines and test materials) to demonstrate the robustness of the method, i.e., the repeatability of the responses to standard NMs. As a further step in this direction, we here reported the results obtained on widely used cell lines, such as A549, exposed to some easily acquired NMs, and unequivocally identified through the newly proposed European Registry of Materials (van Rijn et al., 2021). Eventually, the standardized and validated procedure might be submitted to the OECD as a standard project submission form (SPSF).

In conclusion AB is a reliable test to evaluate the cytotoxicity/proliferation/metabolic response of cells exposed to NMs. Being high throughput makes it an ideal tool to be used on a large scale and in parallel or in combination with other assays e.g. the comet assay for genotoxicity (Azqueta et al., 2022). However, washing steps after exposure and proper controls for possible interference of the NMs with the test need to be always included. The coupling of this metabolism-based test with another class of cytotoxicity method based for example on membrane integrity or cell number is also a major recommendation to strengthen the results.

## Data Availability

The raw data supporting the conclusion of this article will be made available by the authors, without undue reservation.

## References

[B1] Ahmed S. A. Gogal R. M. Walsh J. E. (1994). A new rapid and simple non-radioactive assay to monitor and determine the proliferation of lymphocytes: An alternative to [3H] thymidine incorporation assay. J. Immunol. Methods 170, 211–224. 10.1016/0022-1759(94)90396-4 8157999

[B2] Azqueta A. Stopper H. Zegura B. Dusinska M. Møller P. (2022). Do cytotoxicity and cell death cause false positive results in the *in vitro* comet assay? Mutat. Research/Genetic Toxicol. Environ. Mutagen. 881, 503520. 10.1016/J.MRGENTOX.2022.503520 36031332

[B3] Bonnier F. Keating M. E. Wróbel T. P. Majzner K. Baranska M. Garcia-Munoz A. (2015). Cell viability assessment using the alamar blue assay: A comparison of 2D and 3D cell culture models. Toxicol. Vitro 29, 124–131. 10.1016/J.TIV.2014.09.014 25300790

[B4] Casey A. Herzog E. Davoren M. Lyng F. M. Byrne H. J. Chambers G. (2007). Spectroscopic analysis confirms the interactions between single walled carbon nanotubes and various dyes commonly used to assess cytotoxicity. Carbon N. Y. 45, 1425–1432. 10.1016/J.CARBON.2007.03.033

[B5] Ciappellano S. G. Tedesco E. Venturini M. Benetti F. (2016). *In vitro* toxicity assessment of oral nanocarriers. Adv. Drug Deliv. Rev. 106, 381–401. 10.1016/J.ADDR.2016.08.007 27544694

[B6] Cimpan M. R. Mordal T. Schölermann J. Allouni Z. E. Pliquett U. Cimpan E. (2013). An impedance-based high-throughput method for evaluating the cytotoxicity of nanoparticles. J. Phys. Conf. Ser. 429, 012026. 10.1088/1742-6596/429/1/012026

[B7] Davoren M. Herzog E. Casey A. Cottineau B. Chambers G. Byrne H. J. (2007). *In vitro* toxicity evaluation of single walled carbon nanotubes on human A549 lung cells. Toxicol. Vitro 21, 438–448. 10.1016/J.TIV.2006.10.007 17125965

[B8] Deloid G. M. Cohen J. M. Pyrgiotakis G. Demokritou P. (2017). Preparation, characterization, and *in vitro* dosimetry of dispersed, engineered nanomaterials. Nat. Protoc. 12 (2), 355–371. 10.1038/nprot.2016.172 28102836 PMC5857388

[B9] Dusinska M. Boland S. Saunders M. Juillerat-Jeanneret L. Tran L. Pojana G. (2015). Towards an alternative testing strategy for nanomaterials used in nanomedicine: Lessons from NanoTEST. Nanotoxicology 9, 118–132. 10.3109/17435390.2014.991431 25923349

[B10] Dusinska M. Tulinska J. el Yamani N. Kuricova M. Liskova A. Rollerova E. (2017). Immunotoxicity, genotoxicity and epigenetic toxicity of nanomaterials: New strategies for toxicity testing? Food Chem. Toxicol. 109, 797–811. 10.1016/J.FCT.2017.08.030 28847762

[B11] Elliott J. T. Rösslein M. Song N. W. Toman B. Kinsner-Ovaskainen A. Maniratanachote R. (2017). Toward achieving harmonization in a nanocytotoxicity assay measurement through an interlaboratory comparison study. ALTEX - Altern. animal Exp. 34, 201–218. 10.14573/ALTEX.1605021 27684074

[B12] Fields R. Lancaster M. (1993). Dual-attribute continuous monitoring of cell proliferation/cytotoxicity. Am. Biotechnol. Lab. 11, 48–50. 7763491

[B13] Geys J. Nemery B. Hoet P. H. M. (2010). Assay conditions can influence the outcome of cytotoxicity tests of nanomaterials: Better assay characterization is needed to compare studies. Toxicol. Vitro 24, 620–629. 10.1016/J.TIV.2009.10.007 19850119

[B14] Ghasemi M. Turnbull T. Sebastian S. Kempson I. (2021). The mtt assay: Utility, limitations, pitfalls, and interpretation in bulk and single-cell analysis. Int. J. Mol. Sci. 22, 12827. 10.3390/ijms222312827 34884632 PMC8657538

[B15] Guadagnini R. Moreau K. Hussain S. Marano F. Boland S. (2015). Toxicity evaluation of engineered nanoparticles for medical applications using pulmonary epithelial cells. Nanotoxicology 9, 25–32. 10.3109/17435390.2013.855830 24286383

[B16] Hamid R. Rotshteyn Y. Rabadi L. Parikh R. Bullock P. (2004). Comparison of alamar blue and MTT assays for high through-put screening. Toxicol. Vitro 18, 703–710. 10.1016/J.TIV.2004.03.012 15251189

[B17] Heng B. C. Zhao X. Xiong S. Ng K. W. Boey F. Y. C. Loo J. S. C. (2011). Cytotoxicity of zinc oxide (ZnO) nanoparticles is influenced by cell density and culture format. Arch. Toxicol. 85, 695–704. 10.1007/s00204-010-0608-7 20938647

[B18] Herzog E. Casey A. Lyng F. M. Chambers G. Byrne H. J. Davoren M. (2007). A new approach to the toxicity testing of carbon-based nanomaterials—the clonogenic assay. Toxicol. Lett. 174, 49–60. 10.1016/J.TOXLET.2007.08.009 17920791

[B19] ISO (2018). ISO 19007:2018 - nanotechnologies — *in vitro* MTS assay for measuring the cytotoxic effect of nanoparticles. Available at: https://www.iso.org/standard/63698.html.

[B20] Kasprzak A. Poplawska M. Bystrzejewski M. Grudzinski I. P. (2016). Sulfhydrylated graphene-encapsulated iron nanoparticles directly aminated with polyethylenimine: A novel magnetic nanoplatform for bioconjugation of gamma globulins and polyclonal antibodies. J. Mat. Chem. B 4, 5593–5607. 10.1039/C6TB00838K 32263356

[B21] Kladko D. v. Falchevskaya A. S. Serov N. S. Prilepskii A. Y. (2021). Nanomaterial shape influence on cell behavior. Int. J. Mol. Sci. 22, 5266. 10.3390/IJMS22105266 34067696 PMC8156540

[B22] Méry B. Guy J. B. Vallard A. Espenel S. Ardail D. Rodriguez-Lafrasse C. (2017). *In vitro* cell death determination for drug discovery: A landscape review of real issues. J. Cell Death 10, 1179670717691251. 10.1177/1179670717691251 28469473 PMC5392044

[B23] O’brien J. Wilson I. Orton T. Pognan F. Ë. (2000). Investigation of the Alamar Blue (resazurin) fluorescent dye for the assessment of mammalian cell cytotoxicity. Eur. J. Biochem. 267, 5421–5426. 10.1046/j.1432-1327.2000.01606.x 10951200

[B24] OECD (2018). Guidance document on good *in vitro* method practices (GIVIMP). OECD Ser. Test. Assess. 286. 10.1787/9789264304796-en

[B25] Petersen E. J. Nguyen A. D. Brown J. Elliott J. T. Clippinger A. J. Gordon J. (2021). Characteristics to consider when selecting a positive control material for an *in vitro* assay. ALTEX - Altern. animal Exp. 38, 365–376. 10.14573/ALTEX.2102111 33637998

[B26] Petersen E. J. Uhl R. Toman B. Elliott J. T. Strickland J. Truax J. (2022). Development of a 96-well electrophilic allergen screening assay for skin sensitization using a measurement science approach. Toxics 10, 257. 10.3390/TOXICS10050257 35622670 PMC9147637

[B27] Rampersad S. N. (2012). Multiple applications of alamar blue as an indicator of metabolic function and cellular health in cell viability bioassays. Sensors 12, 12347–12360. 10.3390/s120912347 23112716 PMC3478843

[B28] Riss T. L. Moravec R. A. Niles A. L. Duellman S. Benink H. A. Worzella T. J. (2016). Cell viability assays. Assay Guidance Manual. Available at: https://www.ncbi.nlm.nih.gov/books/NBK144065/.

[B29] Riss T. Niles A. Moravec R. Karassina N. Vidugiriene J. (2019). Cytotoxicity assays: *In vitro* methods to measure dead cells. Assay Guidance Manual. Available at: https://www.ncbi.nlm.nih.gov/books/NBK540958/.

[B30] van Rijn J. Afantitis A. Culha M. Dusinska M. Exner T. Jeliazkova N. (2021). European Registry of materials: Global, unique identifiers for (undisclosed) nanomaterials. Working paper, 10.26434/CHEMRXIV-2021-65894 PMC9400299 36002868

[B31] Worle-Knirsch J. M. Pulskamp K. Krug H. F. (2006). Oops they did it again! Carbon nanotubes hoax scientists in viability assays. Nano Lett. 6, 1261–1268. 10.1021/nl060177c 16771591

